# Performance and Life Prediction of Recycled Concrete Against Sulfate Dry–Wet Cycle Corrosion

**DOI:** 10.3390/ma18102201

**Published:** 2025-05-10

**Authors:** Liangliang Chen, Fufei Wu, Daqing Liu, Chuanteng Huang, Shuang Pu, Jing Wang, Pengfei Luo

**Affiliations:** 1 School of Civil Engineering, Huzhou Vocational and Technical College, Huzhou 313000, China; 2021027@huvtc.edu.cn; 2 Huzhou Key Laboratory of Green Building Technology, Huzhou Vocational and Technical College, Huzhou 313000, China; 3 School of Materials and Architectural Engineering, Guizhou Normal University, Guiyang 550025, China; 4 Zhejiang Zhedong Jietong Road & Bridge Construction Co., Ltd., Ningbo 315000, China; 5 School of Engineering, Zunyi Normal University, Zunyi 563006, China

**Keywords:** recycled concrete, dry–wet cycle, corrosion performance, life prediction

## Abstract

To investigate the sulfate resistance of recycled concrete with composite admixtures under dry–wet cycling, a single-factor experimental design was first conducted to study the deterioration patterns of recycled concrete with single and composite admixtures (ground granulated blast furnace slag (GGBS) and fly ash) under sulfate attack. Based on the single-factor test results, orthogonal experiments were designed with composite admixtures as one influencing factor. Quantitative analysis was performed to determine the impact magnitude and significance of various factors on the sulfate resistance of recycled concrete at different corrosion ages. A damage model for recycled concrete under sulfate dry–wet cycling was established for preliminary service life prediction. The experimental results indicated that the sulfate resistance performance followed the sequence of composite admixtures > single slag admixture > single fly ash admixture. When uncycled (0 cycles), the influence ranking of factors was B (water–binder ratio) > A (recycled coarse aggregate replacement rate) > C (GGBS + fly ash content). After 60 and 120 cycles, the ranking became B > C > A. For the compressive strength regression model, the measured values deviated significantly from the calculated values (−6.88% to 16.66%), while the dynamic elastic modulus model showed good agreement between the measured and calculated values (−2.86% to 4.87%). A three-indicator lifespan prediction equation was established. Using practical engineering parameters (30% recycled aggregate replacement, 0.4 water–binder ratio, 20% fly ash and 20% slag content), the predicted service life of this recycled concrete project was *T* = 117 years. Therefore, incorporating fly ash and slag can effectively improve weak zones in recycled concrete and enhance its durability.

## 1. Introduction

Against the backdrop of accelerating global urbanization and expanding infrastructure construction, concrete, as the most consumed material in the construction industry, has triggered severe ecological crises through its consumption of natural aggregate resources. According to statistics, approximately 40 billion tons of natural sand and gravel are extracted globally each year, equivalent to removing 1270 tons of mineral resources from the Earth’s surface every second [1]. Meanwhile, waste concrete from construction and demolition accounts for 30–40% of municipal solid waste [2]. Landfill disposal of these wastes not only occupies land resources but also generates alkaline leachate that contaminates groundwater. In this context, processing waste concrete into recycled concrete aggregate (RCA) through crushing and screening can achieve over 90% material recycling efficiency in construction [3]. This approach significantly reduces mountain destruction and river ecological imbalance caused by natural aggregate extraction, demonstrating notable resource conservation and carbon reduction benefits [4].

However, the widespread application of recycled aggregate concrete (RAC) faces critical technical challenges. Compared with natural aggregates, internal defects generated during the crushing process substantially alter the material characteristics of recycled coarse aggregates. Scanning electron microscopy analysis reveals that 30–60% of old mortar remains adhered to RCA surfaces [5], resulting in three- to five-times higher water absorption than natural aggregates [6]. X-ray microcomputed tomography studies indicate that the microcrack density within RCA reaches 2.3 times that of natural aggregates [7,8]. These structural defects dramatically increase the complexity of concrete’s three-phase interface (aggregate-old mortar-new mortar), creating multiple weak interfacial transition zones (ITZs). Research has demonstrated that the ITZ thickness in RAC can be 1.5 times greater than in conventional concrete, with the internal porosity increased by 25–40% [9,10], providing rapid transmission channels for corrosive media.

Under harsh environmental conditions, the durability degradation of recycled aggregate concrete (RAC) exhibits accelerated characteristics, with a sulfate attack recognized as one of the most destructive environmental effects. According to the American Concrete Institute (ACI) report, 15% of global concrete structure failures are directly related to a sulfate attack [11]. For RAC, its corrosion process demonstrates unique cross-scale evolution mechanisms. In microscale mass transport, (1) each 1% increase in RAC porosity elevates ion diffusion coefficients by 12–18% [12,13]; (2) electrical potential gradients from Ca^2+^ concentration differences accelerate ion migration in interfacial transition zones (ITZ) [14,15]; (3) crystallization-induced expansion stress from Na_2_SO_4_ solution during wet–dry cycles reaches 12 MPa [16]. In mesoscale chemical reactions, (1) gypsum formation causes 120% volumetric expansion, with XRD analysis showing the gypsum content in RAC reaching 1.8 times that in natural concrete [17]; (2) needle-shaped ettringite crystal growth generates 220% volumetric expansion [18,19]; (3) degradation of C-S-H gel reduces concrete’s binding capacity, decreasing the compressive strength by up to 60% [20,21]. In macroscale performance degradation, Yang et al. [22] observed 50% recycled clay brick aggregates, and 50% recycled concrete aggregates led to a reduction of 6% of the flexural strength compared to the corresponding natural aggregate concrete. Lotfi et al. [23] showed that the reduction of compressive strength for RAC was 10.4%, which was much higher than that of natural aggregate concrete after exposure to freezing (−18 ± 2 °C) and thawing (18 ± 2 °C) with a rate of two to three cycles per day for 100 cycles. Grabiec et al. [24] observed that the recycled concrete aggregate water absorption had a reduction of around 13.5% to 21.2%. Yuncheng Wang et al. [25] developed a fatigue-based damage evolution model for predicting the remaining life of cracked concrete components under cyclic loads. This model, falling within the realm of continuum damage mechanics, can effectively capture the cumulative damage process. Recent studies have highlighted the importance of advanced numerical models in simulating concrete degradation. In prediction models, conventional approaches include Fick’s law-based deterministic model [26], stochastic damage mechanics model [27] and machine learning prediction method [28].

To address these limitations, this study incorporates fly ash and ground granulated blast furnace slag (GGBS) individually and compositely into RAC. It systematically investigates their modification effects on sulfate resistance and establishes a three-indicator service life prediction equation through single-factor experimental design, ultimately achieving lifespan prediction for optimized RAC systems.

## 2. Materials and Methods

### 2.1. Experimental Materials

The cement used was P.O 42.5R grade ordinary Portland cement (produced by the Xinjiang Tianshan Cement Plant, Urumqi, China) with qualified soundness. It has a specific surface area of 380 m^2^/kg, standard consistency water demand of 28% and initial and final setting times of 180 min and 210 min, respectively. The flexural strengths at 3 d and 28 d were 5.3 MPa and 8.7 MPa, while the compressive strengths at 3 d and 28 d were 26.5 MPa and 53.7 MPa, respectively. Class II fly ash from the Urumqi Power Plant show a loss on ignition of 1.26%, fineness of 4.6%, water demand ratio of 94% and density of 2.5 g/cm^3^. The ground granulated blast furnace slag (GGBS) was from Xinjiang Baoxin Shengyuan Building Materials Co., Ltd., Urumqi, China, with loss on ignition of 0.2%, water demand ratio of 92% and density of 2.9 g/cm^3^. The chemical composition of fly ash and GGBS provided by the manufacturer is presented in Table 1. The washed sand had a fineness modulus of 3.5 with particle size distribution in zone I. The mixing/curing water was laboratory tap water, and a naphthalene-based high-range water reducer was used. Both mixing and curing water met the drinking water standards. The particle sizes of natural pebbles ranged from 5 to 20 mm, the bulk density was 1437 kg/m^3^, the apparent density was 2735 kg/m^3^, the water absorption rate was 0.21% and the crushing index was 4.94%. The particle size range of recycled aggregates was 5–20 mm, the bulk density was 1311 kg/m^3^, the apparent density was 2486 kg/m^3^, the water absorption rate was 3.83% and the crushing index was 13.72%. It was important to acknowledge the potential biases that could have influenced the results of this study. In terms of selection bias, although efforts were made to select representative materials from common sources in the construction industry, the variability in material properties from different regions and production batches might not have been fully captured. To minimize this bias, the materials used in the experiment, such as cement, fly ash and recycled aggregates, were sourced from reliable manufacturers with consistent quality control. However, future research could expand the range of material sources to further validate the findings.

### 2.2. Experimental Program

The experiment utilized two specimen types: 100 mm inside the cube and 100 mm × 100 mm × 400 mm prisms, for determining the cube compressive strength and dynamic elastic modulus, respectively. Specimens were prepared according to mix proportions (as shown in Table 2) and subjected to standard curing for 28 days before sulfate wet–dry cycle corrosion testing. The wet–dry cycling tests were conducted using an MKS-54B concrete sulfate wet–dry cycle testing machine (Beijing Shourui Co., Ltd., Beijing, China) in compliance with GB/T50082-2009 [29] Each 24-h cycle comprised:

14 h of immersion in 5% Na_2_SO_4_ solution,

1 h of air-drying,

6 h of baking (80 °C),

2 h of air-cooling,

1 h of baking (35 °C).

### 2.3. Testing Methods and Data Processing

**Figure 1:**
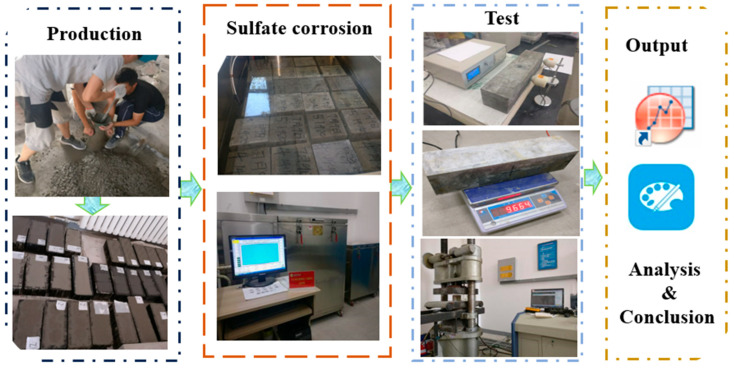
The flow chart of the research.

Referring to a large amount of previous research literature [30,31], the instruments used in the experiment have been widely applied and recognized in the field of concrete material performance studies. The instruments for measuring the compressive strength and dynamic elastic modulus of concrete are based on mature mechanical theories and have been proven to accurately measure these physical quantities in numerous similar studies. They align well with the theoretical framework for evaluating the performance of recycled concrete in this study, demonstrating good structural validity. The preparation of recycled concrete, the sulfate wet–dry cycling process and parameter measurements are illustrated in Figure 1. After that, the cube compressive strength, dynamic elastic modulus and mass loss rate were measured at 0, 60 and 120 cycles.

#### 2.3.1. Cube Compressive Strength

Cube compressive strength was measured at 0, 60 and 120 cycles by a YE-200A 200-ton hydraulic pressure testing machine manufactured by the Hongshan Testing Machine Factory (Dongguan, China), and the testing procedure was as follows:

(1) Before testing, wipe the surface moisture of the test block with a dry cloth and cover it with a cloth to prevent excessive drying.

(2) Clean any residual debris from the pressure-bearing plate of the testing machine with a dry cloth.

(3) Place the test block in the center of the lower pressure plate, start the testing machine and apply a continuous, uniform load at a rate of 0.3 MPa/s to 0.5 MPa/s until the test block fails. Stop loading at this point and record the failure load.

The formula for calculating the compressive strength of the recycled concrete cube was shown in Equation (1).

(1) $$f_{cu}=\frac{P}{A}$$

where *f_cu_* was the compressive strength (MPa), *P* was the failure load (N) and *A* was the bearing area of the test block (mm^2^).

#### 2.3.2. Dynamic Elastic Modulus

The dynamic elastic modulus was measured using a DT-16 Dynamic Elastic Modulus Tester (Tianjin Yaxing automation experimental instrument factory, Tianjin, China). This device calculates the dynamic elastic modulus of recycled concrete by determining the resonant frequency of the test specimen.

(2) $$E_{d}=9.46\times 10^{-4}\times \frac{m\cdot l^{3}\cdot f^{2}}{a^{4}}\times K$$

where *E_d_* was the dynamic elastic modulus of recycled concrete (MPa), *a* was the side length of the square cross-section (mm), *l* was the length of the specimen (mm), *m* was the reference mass of the test specimen before corrosion (kg), *f* was the fundamental vibration frequency in transverse mode (Hz) and *K* was the correction factor (dimensionless); When *l*/*α* = 3, 4 and 5, *K* was 1.68, 1.40 and 1.26, respectively.

#### 2.3.3. Mass Loss Rate

The mass was measured using a JSB15-1 electronic balance (Shanghai Puchun Measuring Instrument Co., Ltd., Shanghai, China), and the mass loss was then calculated.

(3) $$∆m=\frac{m-m_{n}}{m}$$

where Δ*m* was the mass loss rate after *n* wet–dry cycles (%), and *m_n_* was the mass of the specimen after *n* wet–dry cycles (kg).

#### 2.3.4. Data Processing

For all compressive strength, dynamic elastic modulus and mass loss rate evaluations, five specimens were tested per condition. The reported value represents the mean average of the three samples, with all individual datapoints required to fall within ±10% of the mean to ensure statistical validity.

## 3. Results and Discussion

### 3.1. The Influence Law of Single and Compound Doping

**Figure 2:**
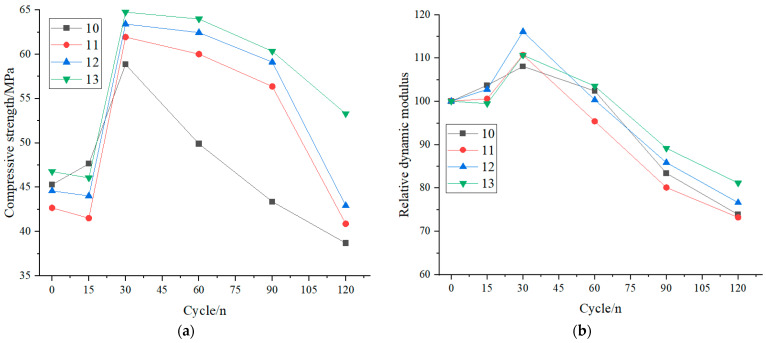
Effect of recycled aggregate replacement rate on the compressive strength and relative dynamic modulus: (**a**) compressive strength and (**b**) relative dynamic modulus.

Figure 2 illustrates the effects of GGBS and fly ash on the compressive strength and relative dynamic elastic modulus of recycled concrete under sulfate wet–dry cycling corrosion. Recycled concrete incorporating GGBS and fly ash exhibited a three-stage evolution pattern during sulfate attack: initial reduction or stabilization, followed by growth and eventual gradual decline (slowest in the third stage). Hongru Zhang et al. [30] studied the performance evolution of recycled aggregate concrete under sulfate attacks in different exposure conditions. They found that the mechanical properties of RAC, such as compressive strength and dynamic elastic modulus, also showed a trend of initial increase or stabilization, followed by a decrease over time. Vertically, throughout the corrosion process, specimens with binary GGBS–fly ash blending demonstrated higher compressive strength and relative dynamic elastic modulus than those with single GGBS blending under equal dosage, while single GGBS blending outperformed single fly ash blending. Notably, GGBS and fly ash admixed recycled concrete exhibited superior corrosion resistance compared to the non-admixed counterparts, regardless of blending type.

This phenomenon can be primarily attributed to the low reactivity of GGBS and fly ash, which require alkaline activation to initiate hydration. Relatively speaking, the activity of GGBS is higher than that of fly ash, and its compressive strength is relatively high. After GGBS and fly ash are mixed, the superposition effect can be exerted, and its compressive strength can be increased; thus, the compressive strength values are higher than the other groups, after curing for 28 days. During early cycling (15 cycles), the reaction of the remaining portlandite with sulfate ions and C-A-H that forms ettringite in the pores, filling them first and increasing at the beginning the compressive strength. Over time, portlandite from cement hydration provided the necessary alkaline environment, triggering substantial secondary hydration of GGBS and fly ash. This generated C-S-H gel, resulting in significant strength and modulus increases at 15–30 cycles. Beyond 30 cycles (up to 120 cycles), sulfate induced physical-chemical actions, because of an excess of formed ettringite, led to a decrease of the compressive strength due to the expansive action of ettringite and samples’ cracking. These mechanisms collectively explain the triphasic pattern. The initial strength reduction with higher GGBS and fly ash content stems from their delayed hydration and reduced cement proportion. Early strength development relies predominantly on cement-derived C-S-H gel. However, alkali-activated GGBS and fly ash progressively contributed to C-S-H formation during the mid to late stages (15–120 cycles), enhancing performance proportionally to their dosage. The superiority of binary blending over single GGBS blending, and GGBS over fly ash, arises from three factors.

Particle gradation: GGBS particles < fly ash particles < cement particles, enabling mutual filling for a denser microstructure.

Reactivity hierarchy: Fly ash reactivity < GGBS reactivity < cement reactivity, ensuring complementary hydration kinetics.

Strength synergy: Combined hydration products from blended systems create a more robust and durable matrix than single-admixture systems.

### 3.2. Results Analysis of the Orthogonal Test

#### 3.2.1. Extreme Range Analysis

To better evaluate the influence of various factors on the sulfate wet–dry cycling corrosion resistance of recycled concrete at different ages, as well as the evolution of optimal and inferior parameter combinations, range analysis was conducted based on measured indices at 0, 60 and 120 cycles. The range analysis results for cube compressive strength are summarized in Table 3, and those for the dynamic elastic modulus are presented in Table 4.

Table 3 and Table 4 present the range values (R) of compressive strength and the dynamic elastic modulus for recycled concrete under sulfate wet–dry cycling corrosion at 0, 60 and 120 cycles, respectively. A larger R value indicates a greater influence of the corresponding factor. The analysis reveals the order of factor influence on compressive strength at 0 cycles: B > A > C, at 60 cycles: B > C > A and at 120 cycles: B > C > A. Order of factor influence on the dynamic elastic modulus is, at 0 cycles: B > A > C, at 60 cycles: B > C > A and at 120 cycles: B > C > A. This confirms that the water-to-binder ratio (B) was the dominant influencing factor throughout the sulfate corrosion process. Notably, at 0 cycles: a recycled coarse aggregate replacement rate (A) exhibits greater influence than blended mineral admixture content (C) at 60 and 120 cycles: blended GGBS and fly ash content (C) surpasses the recycled coarse aggregate replacement rate (A) in influence magnitude.

#### 3.2.2. Variance Analysis

Univariate analysis of variance (ANOVA) was conducted using SPSS software (SPSS 29) to analyze the cube compressive strength and dynamic elastic modulus of recycled concrete after 0, 60 and 120 cycles of sulfate dry–wet corrosion. According to the ANOVA results in Table 5, the variation in the water-to-binder ratio had the most significant and highly significant effect on all evaluation indicators throughout the sulfate dry–wet corrosion process. At 0 cycles of sulfate dry–wet corrosion, the significance of the water-to-binder ratio’s effect on all indicators was greater than that of the recycled coarse aggregate replacement rate, which, in turn, had a more significant effect than the composite mineral admixture content. At 60 and 120 cycles of sulfate dry–wet corrosion, the order of significance shifted. The water-to-binder ratio remained the most influential factor, followed by the composite admixture content, with the recycled coarse aggregate replacement rate showing the least significant effect. Compared to the cube compressive strength, variations in factor levels had a more pronounced impact on the dynamic elastic modulus. This indicates that the dynamic elastic modulus was a more sensitive evaluation indicator for assessing the resistance of recycled concrete to sulfate dry–wet corrosion.

#### 3.2.3. Regression Analysis

The experimental results for cube compressive strength and dynamic elastic modulus of recycled concrete under 0, 60 and 120 cycles of sulfate dry–wet corrosion were subjected to multiple linear regression analysis. The initial multiple linear regression model was postulated as Equation (4):

*y_i_* = *a*_0_ + *a*_1_*x*_1_ + *a*_2_*x*_2_ + *a*_3_*x*_3_ (4)

where *y_i_* = cube compressive strength or dynamic elastic modulus; *x*_1_ = recycled coarse aggregate replacement ratio; *x*_2_ = water-to-binder ratio, *x*_3_ = blended mineral admixture content and *a_0_*, *a*_1_, *a*_2_ and *a*_3_ = regression parameters (aging influence coefficients under dry–wet cycles). By substituting the evaluation index results from Table 3 and Table 4 (measured at 0, 60 and 120 corrosion cycles) into Equation (4), the multiple linear regression equations for different aging periods were derived, as presented in Table 6.

Table 6 demonstrates that all regression equations exhibit correlation coefficients exceeding 0.8, confirming the statistical significance and validity of the fits. Analysis of the regression parameters reveals that, at 0 corrosion cycles: regression parameters *a*_1_, *a*_2_ and *a*_3_ for both compressive strength and the elastic modulus are negative (*a*_1_ < 0, *a*_2_ < 0 and *a*_3_ < 0), indicating these properties decrease with the increasing recycled aggregate replacement ratio, water-to-binder ratio and mineral admixture content. The absolute parameter magnitudes follow |*a*_2_| > |*a*_1_| > |*a*_3_|, signifying that the water-to-binder ratio exerts the strongest influence on corrosion resistance, followed by recycled aggregate replacement ratio, with the mineral admixture content showing the least impact. At 60/120 corrosion cycles: parameters transition to *a*_1_ < 0, *a*_2_ < 0 and *a*_3_ > 0, suggesting compressive strength and elastic modulus now increase with the mineral admixture content while remaining inversely proportional to the recycled aggregate replacement and water-to-binder ratios. Magnitude hierarchy shifts to |*a*_2_| > |*a*_3_| > |*a*_1_|, highlighting the water-to-binder ratio as the dominant factor, followed by the mineral admixture content, with recycled aggregate replacement showing a reduced influence. These findings align consistently with both the range analysis and ANOVA conclusions, validating the robustness of the regression models.

#### 3.2.4. Regression Model Verification

To validate the reliability of the established compressive strength and dynamic elastic modulus regression models, the measured values of these properties under 0, 60 and 120 sulfate dry–wet corrosion cycles were substituted into the regression equations in Table 6. A comparative analysis between the calculated and measured values was conducted using relative error (RE) as the quantitative metric for model accuracy. Lower RE values indicate higher precision. The RE formula was defined in Equation (5):

(5) δ=Δ/L×100%

where *δ*: relative error (%), Δ: absolute error (calculated value–measured value) and *L*: true value (measured value). The validation results and error analysis are summarized in Table 7.

Key Observations from Table 7: Compressive Strength fit: 0 cycles: RE ranges from −14.87% to 22.77%, 60 cycles: RE ranges from −6.88% to 16.66% and 120 cycles: RE ranges from −8.51% to 14.80%. Significant deviations between the calculated and measured values indicate low reliability of the compressive strength regression fit. Further experimental refinement was required to improve accuracy. Dynamic Elastic Modulus fit: 0 cycles: RE ranges from −2.61% to 3.66%, 60 cycles: RE ranges from −1.72% to 2.06% and 120 cycles: RE ranges from −2.86% to 4.87%. Close agreement between the calculated and measured values confirms reasonable reliability of the dynamic elastic modulus regression fit. This fit demonstrates practical applicability for assessing recycled concrete performance under sulfate dry–wet corrosion.

### 3.3. Determination of Influence Function

#### 3.3.1. Determination of Influence Function of the Replacement Rate of Recycled Coarse Aggregate

**Figure 3:**
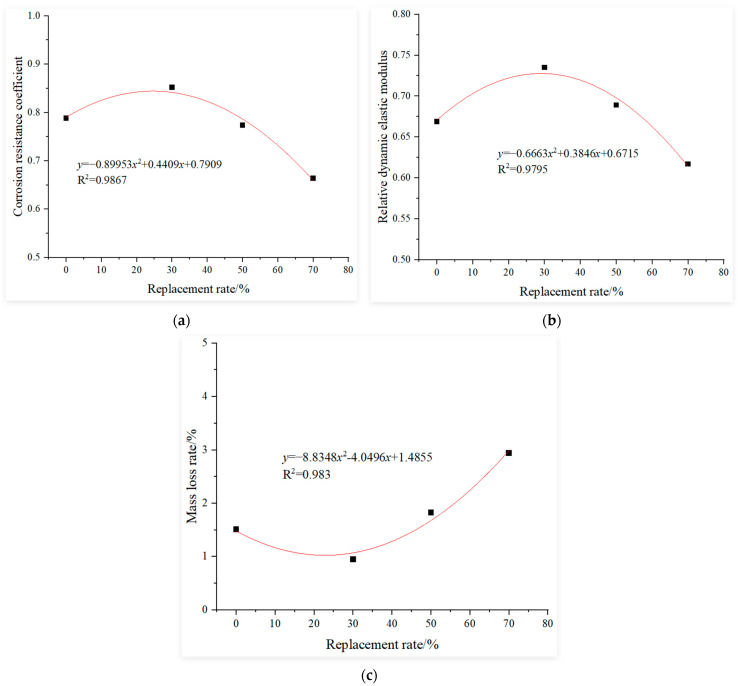
Influence function of the replacement rate: (**a**) corrosion resistance coefficient, (**b**) relative dynamic elastic modulus and (**c**) mass loss rate.

This study established benchmark values using the test results of recycled concrete with 0% recycled coarse aggregate replacement rate after 120 sulfate wet–dry cycles. Regression analysis was performed on test data from recycled concrete specimens with a constant water-to-binder ratio of 0.4, 0% fly ash and GGBS contents and recycled coarse aggregate replacement rates of 0%, 30%, 50% and 70% subjected to 120 sulfate cycles. The fitted curves of the influence functions for the recycled aggregate replacement rate were derived using three evaluation metrics under sulfate corrosion: compressive corrosion resistance coefficient, relative dynamic elastic modulus and mass loss rate. These curves are presented in Figure 3.

#### 3.3.2. Determination of the Water–Binder Ratio Influence Function

**Figure 4:**
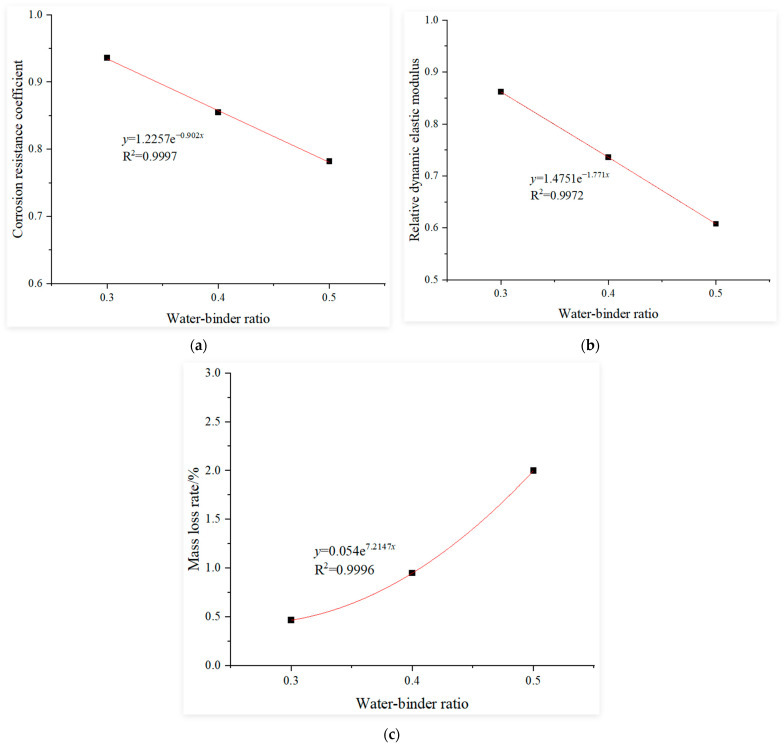
Influence function of the water–binder ratio: (**a**) corrosion resistance coefficient, (**b**) relative dynamic elastic modulus and (**c**) mass loss rate.

This study uses the experimental results of recycled concrete with a water-to-binder ratio of 0.3 after 120 cycles of sulfate dry–wet corrosion as the baseline. Regression analysis was conducted on the experimental results of recycled concrete under the following conditions after 120 cycles of sulfate dry–wet corrosion: constant replacement rate of recycled coarse aggregate at 30%; fly ash content and GGBS content both maintained at 0% and water-to-binder ratios of 0.3, 0.4 and 0.5. The fitting curves for the influence functions of the water-to-binder ratio under sulfate dry–wet corrosion using a compressive corrosion resistance coefficient, relative dynamic elastic modulus and mass loss rate as the evaluation indices, are shown in Figure 4.

#### 3.3.3. Determination of the Influence Function of Fly Ash Content

**Figure 5:**
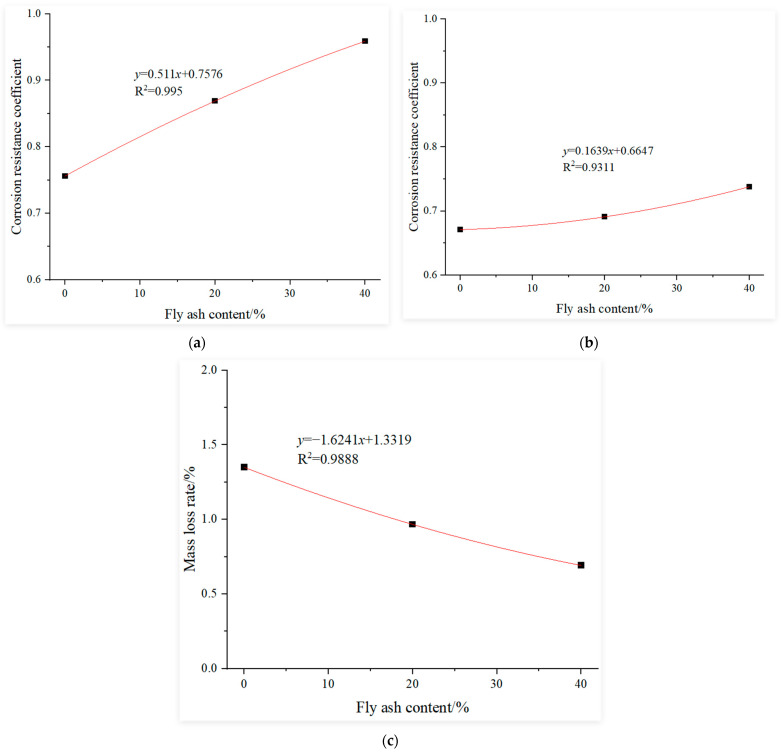
Influence function of the fly ash content: (**a**) corrosion resistance coefficient, (**b**) relative dynamic elastic modulus and (**c**) mass loss rate.

This study uses the experimental results of recycled concrete with 0% fly ash content after 120 cycles of sulfate dry–wet corrosion as the baseline. Regression analysis was conducted on the experimental results of recycled concrete under the following conditions after 120 cycles of sulfate dry–wet corrosion: constant replacement rate of recycled coarse aggregate at 30%; constant water-to-binder ratio at 0.4; constant slag content at 0% and fly ash content of 0%, 20% and 40%. The fitting curves for the influence functions of the fly ash content under sulfate dry–wet corrosion, using the compressive corrosion resistance coefficient, relative dynamic elastic modulus and mass loss rate as the evaluation indices, are shown in Figure 5.

#### 3.3.4. Determination of Influence Function of the GGBS Content

**Figure 6:**
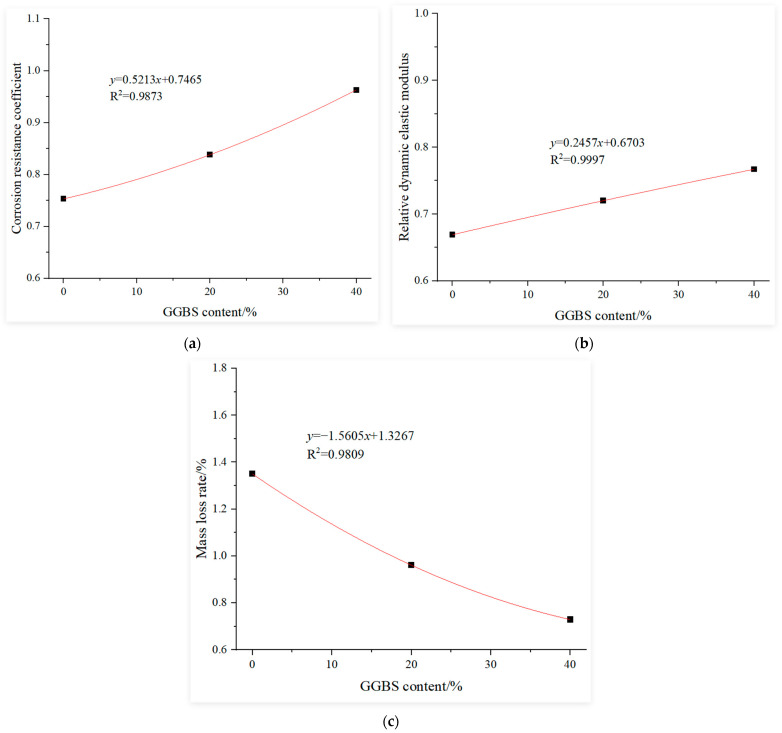
Influence function of the GGBS content: (**a**) corrosion resistance coefficient, (**b**) relative dynamic elastic modulus and (**c**) mass loss rate.

This study uses the experimental results of recycled concrete with 0% slag content after 120 cycles of sulfate dry–wet corrosion as the baseline. Regression analysis was conducted on the experimental results of recycled concrete under the following conditions after 120 cycles of sulfate dry–wet corrosion: constant replacement rate of recycled coarse aggregate at 30%; constant water-to-binder ratio at 0.4; constant fly ash content at 0% and slag content of 0%, 20% and 40%. The fitting curves for the influence functions of the slag content under sulfate dry–wet corrosion, using a compressive corrosion resistance coefficient, relative dynamic elastic modulus and mass loss rate as evaluation indices, are shown in Figure 6.

### 3.4. Establish Life Prediction Model Based on the Single-Factor Design

#### 3.4.1. Proposal of the Service Life Prediction Model

Theoretically, the service life of concrete was infinite. However, due to various environmental factors, its lifespan was significantly shortened. For example, under sulfate dry–wet cyclic corrosion, the degradation of concrete was caused by internal structural damage. According to the material decay equation theory [28], all real-world materials undergo decay, and recycled concrete was no exception. First, assuming the initial value of a durability indicator for recycled concrete was *X*_0_, and *X_t_* represents the value of the indicator after decay at time *t* [30]. The decay rate expression for recycled concrete was given by Equation (6):

(6) $$\frac{dX_{t}}{dt}=-\lambda (X_{t}-X_{0})$$

Integrating Equation (6) yields Equation (7):

(7) $$f=\frac{X_{t}}{X_{o}}=\alpha e^{-\lambda t}$$

The mass loss rate can be calculated using Equation (8):

(8) $$f\prime =\frac{X_{o}-X_{t}}{X_{o}}=1-\frac{X_{t}}{X_{o}}=1-\alpha e^{-\lambda t}$$

In these equations: λ was the decay coefficient. α was an undetermined coefficient, representing the influence coefficients of recycled coarse aggregate replacement rate, water-to-binder ratio, fly ash content and GGBS content. *X*_0_ and *X_t_* were the values of the evaluation indicator at 0 cycles and *t* cycles of sulfate dry–wet corrosion, respectively.

This experiment focuses on the following influencing factors: recycled coarse aggregate replacement rate (*R*), water-to-binder ratio (*W*), fly ash content (*F*) and GGBS content (*S*). The service life of recycled concrete was predicted based on the cubic compressive corrosion resistance coefficient, relative dynamic elastic modulus and mass loss rate.

When *f_R_* = *f_W_* = *f_F_* = *f_S_* = *f*, Equation (9) was derived:

(9) $$t=-\frac{\ln\frac{f^{4}}{\alpha }}{\lambda }$$

#### 3.4.2. Determination of Natural Decay Coefficient

According to the national standard, GB/T50082-2009, the durability life of concrete under sulfate dry–wet cyclic corrosion was considered terminated when the compressive strength after *t* cycles falls below 75% of the initial compressive strength (0 cycles), the relative dynamic elastic modulus drops to 60%, and the mass loss rate exceeds 4%. For recycled concrete under sulfate dry–wet corrosion, this standard was also adopted. Based on extensive research, scholars widely agree on the following definitions: Standard service life [31]: time elapsed when the relative dynamic elastic modulus decays to 60%, calculated by Equation (10):

(10) $$T_{1}=-\frac{1}{\lambda }\ln0.6$$

Half-life service life: time elapsed when the relative dynamic elastic modulus decays to 50%, calculated by Equation (11):

(11) $$T_{2}=-\frac{1}{\lambda }\ln0.5$$

Mean service life: time elapsed when the relative dynamic elastic modulus decays to 1/*e* ≈ 36.78%, calculated by Equation (12):

(12) $$T_{3}=-\frac{1}{\lambda }\ln0.3678$$

In practical engineering, the structures most vulnerable to sulfate dry–wet corrosion include dams, bridges and tunnels, which typically have a design service life of 100 years [32]. Therefore, this study assumes a standard service life *T* = 100 years for recycled concrete when the relative dynamic elastic modulus reaches 60%. Substituting into Equation (10), the decay coefficient was determined as λ = 0.005. Since the decay patterns of the compressive corrosion resistance coefficient and relative dynamic elastic modulus are similar, the same decay coefficient λ applies. Using Equation (7), the decay equations for the compressive corrosion resistance coefficient and relative dynamic elastic modulus (excluding influencing factors) are derived as Equation (13):

(13) $$f_{0}=e^{-0.005t}$$

For the mass loss rate, the durability life was considered terminated when the mass loss rate reached 4%. Following the same method, the decay coefficient for the mass loss rate was determined as $\lambda^{\prime }$ = 0.0004. However, according to the literature [33,34,35], the decay coefficient for conventional concrete was λ ≥ 0.002. Thus, this study adopted λ = 0.002 for recycled concrete based on the mass loss rate. Substituting into Equation (8), the decay equation for the mass loss rate (excluding influencing factors) was given by Equation (14):

(14) $$f_{0}^{\prime }=1-e^{-0.002t}$$

### 3.5. Establishment of Life Prediction Equation Based on Three Indices

#### 3.5.1. Life Prediction Model Establishment

It was assumed that SO_4_^2−^ in the erosive environment penetrates into recycled concrete through diffusion, following the decay law of substances, while simultaneously causing physicochemical damage. By substituting the previously derived influence functions into the decay equations, a service life prediction model for recycled concrete under sulfate dry–wet cyclic corrosion can be established for each evaluation indicator.

For the compressive corrosion resistance coefficient, substituting into Equation (7) yields $f_{R}=\alpha_{R}e^{-\lambda t}$, $f_{W}=\alpha_{W}e^{-\lambda t}$, $f_{F}=\alpha_{F}e^{-\lambda t}$ and $f_{S}=\alpha_{S}e^{-\lambda t}$.

When $f_{R}=f_{W}=f_{F}=f_{S}$, substituting into Equation (9) obtains the service life prediction model based on the compressive corrosion resistance coefficient, as shown in Equation (15):

(15) $$t=-\frac{\ln\frac{f^{4}}{\alpha_{R}\alpha_{W}\alpha_{F}\alpha_{S}}}{0.005}$$

For the relative dynamic elastic modulus, substituting into Equation (7) yields $f_{R}^{\prime }=1-\alpha_{R}^{\prime }e^{-\lambda t}$, $f_{W}^{\prime }=\alpha_{W}^{\prime }e^{-\lambda t}$, $f_{F}^{\prime }=\alpha_{F}^{\prime }e^{-\lambda t}$ and $f_{S}^{\prime }=\alpha_{S}^{\prime }e^{-\lambda t}$.

When $f_{R}^{\prime }=f_{W}^{\prime }=f_{F}^{\prime }=f_{S}^{\prime }$, substituting into Equation (9) obtains the service life prediction model based on the relative dynamic elastic modulus, as shown in Equation (16):

(16) $$t^{\prime }=-\frac{\ln\frac{f^{\prime 4}}{\alpha_{R}^{\prime }\alpha_{W}^{\prime }\alpha_{F}^{\prime }\alpha_{S}^{\prime }}}{0.005}$$

For the mass loss rate, substituting into Equation (8) yields $f_{R}^{\prime \prime }=1-\alpha_{R}e^{-\lambda t}$, $f_{W}^{\prime \prime }=\alpha_{W}e^{-\lambda t}$, $f_{F}^{\prime \prime }=\alpha_{F}e^{-\lambda t}$ and $f_{S}^{\prime \prime }=\alpha_{S}e^{-\lambda t}$.

When $f_{R}^{\prime \prime }=f_{W}^{\prime \prime }=f_{F}^{\prime \prime }=f_{S}^{\prime \prime }$, substituting into Equation (9) obtains the service life prediction model based on the mass loss rate, as shown in Equation (17):

(17) $$t^{\prime \prime }=-\frac{\ln\frac{{\left(1-f^{\prime \prime }\right)}^{4}}{\alpha_{R}^{\prime \prime }\alpha_{W}^{\prime \prime }\alpha_{F}^{\prime \prime }\alpha_{S}^{\prime \prime }}}{0.002}$$

By combining Equations (15)–(17), the service life prediction model for recycled concrete under sulfate dry–wet cyclic corrosion was established, as shown in Equation (18):

(18) $$T=\frac{1}{3}(t+t^{\prime }+t^{\prime \prime })=-\frac{1}{3}(\frac{\ln\frac{f^{4}}{\alpha_{R}\alpha_{W}\alpha_{F}\alpha_{S}}}{0.005}+\frac{\ln\frac{f^{\prime 4}}{\alpha_{R}^{\prime }\alpha_{W}^{\prime }\alpha_{F}^{\prime }\alpha_{S}^{\prime }}}{0.005}+\frac{\ln\frac{{\left(1-f^{\prime \prime }\right)}^{4}}{\alpha_{R}^{\prime \prime }\alpha_{W}^{\prime \prime }\alpha_{F}^{\prime \prime }\alpha_{S}^{\prime \prime }}}{0.002})$$

where:

$$\begin{array}{c} \alpha_{r}=-0.8953r^{2}+0.4409r+0.7909,\;\alpha_{r}^{\prime }=-0.6663r^{2}+0.3846r+0.6715\\ \alpha_{r}^{\prime \prime }=8.8348r^{2}-4.0496r+1.4855,\;\alpha_{w}=1.2257e^{-0.902w},\\ \alpha_{w}^{\prime }=1.4751e^{-1.771w},\;\alpha_{w}^{\prime \prime }=0.054e^{7.2147w}\\ \alpha_{f}=0.511f+0.7576,\;\alpha_{f}^{\prime }=0.1639f+0.6647\\ \alpha_{f}^{\prime \prime }=-1.6241f+1.3319,\;\alpha_{s}=0.5213s+0.7465\\ \alpha_{s}^{\prime }=0.2457s+0.6703,\;\alpha_{s}^{\prime \prime }=-1.5605s+1.3267 \end{array}$$

Incorporating these three indicators into one model aims to provide a more comprehensive assessment of the comprehensive durability of recycled concrete in a complex corrosion environment. In real-world engineering, the failure of concrete was often the result of the combined action of multiple performance changes.

When establishing this combined model, we made several assumptions. First, we assumed that these three indicators are equally important in evaluating the durability of recycled concrete. Thus, they are given the same weight in the model. However, in practical engineering, the sensitivity of different structures to each indicator may vary. Second, we assumed that the sulfate penetration and corrosion processes in the corrosive environment are relatively stable and uniform, without significant influence from other complex factors such as local environmental differences and construction defects. In actual projects, these factors can cause non-uniformity in the corrosion process, affecting the accuracy of the model.

The limitations of this model are mainly reflected in the following aspects. On the one hand, the model does not fully consider the diversity and complexity of various factors in different regions and engineering environments. For example, differences in water quality and soil composition in different regions can lead to variations in the degree and mechanism of sulfate corrosion, for which the model was difficult to fully account. On the other hand, the model was established based on experimental data, and there may be certain differences between the experimental conditions and actual engineering situations. This may result in prediction errors when the model is applied in practice.

#### 3.5.2. Case Study

A practical engineering project uses recycled concrete structures in a sulfate dry–wet cyclic corrosion environment. The design strength of the recycled concrete was C40, with the following parameters: replacement rate of recycled coarse aggregate: 30%, water-to-binder ratio: 0.4, fly ash content: 20% and GGBS content: 20%. The service life of the recycled concrete structure was considered terminated when any of the following conditions were met: compressive corrosion resistance coefficient ≤ 75%, relative dynamic elastic modulus ≤ 60% and mass loss rate ≥ 4%. Substituting the above parameters into the service life prediction in Equation (18), the predicted service life of the recycled concrete structure was *T* = 117 years.

## 4. Conclusions

It was important to note that the findings of this study are based on specific experimental conditions. it should be emphasized that these findings cannot be directly applied to all concrete structures or different corrosion environments without further verification and adjustment. However, the research results have significant implications for the design, construction and maintenance of recycled concrete structures in environments with sulfate-rich conditions, such as coastal areas and regions near saline–alkali lands. According to this experiment, more complex multifactor coupling models can be established in future research. Special studies on the sulfate resistance of recycled concrete can be carried out for different special engineering scenarios.

(1) At the same admixture content, the sulfate resistance of composite slag and fly ash admixtures > single slag admixture > single fly ash admixture.

(2) At 0 dry–wet cycles, the influence ranking of factors was B (water–binder ratio) > A (recycled coarse aggregate replacement rate) > C (composite admixture content). After 60 and 120 cycles, the ranking shifted to B > C > A. Throughout the sulfate dry–wet cycling process, the water–binder ratio remained the dominant influencing factor.

(3) At 0 cycles, the significance of the influencing factors followed the water–binder ratio > recycled coarse aggregate replacement rate > composite admixture content. After 60 and 120 cycles, the significance order became: water–binder ratio > composite admixture content > recycled coarse aggregate replacement rate. Throughout the corrosion process, the water–binder ratio exhibited the most significant and highly pronounced impact.

(4) For the compressive strength regression model, significant deviations existed between the measured and calculated values (−6.88% to 16.66%), necessitating extensive experimental studies to improve the accuracy. In contrast, the dynamic elastic modulus regression model showed strong agreement between the measured and calculated values (−2.86% to 4.87%), demonstrating the reference value and applicability for evaluating recycled concrete under sulfate dry–wet cycling.

(5) Based on material degradation laws and single-factor test results, a lifespan prediction model was established using a compressive corrosion resistance coefficient, relative dynamic elastic modulus and mass loss as evaluation indicators. Applied to practical engineering parameters, the service life of the recycled concrete project was predicted as *T* = 117 years.

## Figures and Tables

**Table 1:** Chemical composition of fly ash and GGBS.

Name	SiO_2_	Al_2_O_3_	Fe_2_O_3_	CaO	MgO	SO_3_
Fly ash/wt.%	56.17	20.96	6.05	5.86	2.49	1.13
GGBS/wt.%	35.43	16.77	0.84	35.59	1.95	0.20

**Table 2:** Mix proportion design of the recycled concrete.

Category	No.	Replacement Rate of Recycled Aggregate (A)/%	Water–Binder Ratio (B)	Content of GGBS + Fly Ash (C)/%	The Amount of Component Materials in Concrete/(kg m^−3^)							
					Cement	Fly Ash	GGBS	Water	Sand	RCA	Natural Stone	Water Reducing Agent
Orthogonal test group	1	1 (30)	1 (0.3)	1 (0 + 0)	400	0	0	120	837	307	716	4.0
	2	1 (30)	2 (0.4)	2 (10 + 10)	240	80	80	160	764	307	716	2.8
	3	1 (30)	3 (0.5)	3 (20 + 20)	80	160	160	200	712	307	716	2.0
	4	2 (50)	1 (0.3)	2 (10 + 10)	160	80	160	120	837	512	512	4.0
	5	2 (50)	2 (0.4)	3 (20 + 20)	240	160	0	160	764	512	512	2.8
	6	2 (50)	3 (0.5)	1 (0 + 0)	320	0	80	200	712	512	512	2.0
	7	3 (70)	1 (0.3)	3 (20 + 20)	160	160	80	120	837	716	307	4.0
	8	3 (70)	2 (0.4)	1 (0 + 0)	240	0	160	160	764	716	307	2.8
	9	3 (70)	3 (0.5)	2 (10 + 10)	320	80	0	200	712	716	307	2.0
Single and compound doping test group	10	30	0.4	(0 + 0)	400	0	0	400	160	307	716	2.8
	11	30	0.4	(0 + 40)	240	160	0	240	160	307	716	2.8
	12	30	0.4	(40 + 0)	240	0	160	240	160	307	716	2.8
	13	30	0.4	(20 + 20)	240	80	80	240	160	307	716	2.8

**Table 3:** Extreme range analysis of compressive strength.

No.	Orthogonal Factor				0 Cycles of Corrosion/MPa	60 Cycles of Corrosion/MPa	120 Cycles of Corrosion/MPa
	A	B	C	D (Empty)			
1	30%	0.3	0%	1	66.73	57.95	44.04
2	30%	0.4	20%	2	48.55	51.07	36.92
3	30%	0.5	40%	3	39.68	48.65	39.82
4	50%	0.3	20%	3	55.53	60.29	45.82
5	50%	0.4	40%	1	49.88	53.22	43.30
6	50%	0.5	0%	2	51.09	38.40	29.18
7	70%	0.3	40%	2	41.86	58.02	46.00
8	70%	0.4	0%	3	44.31	34.02	25.86
9	70%	0.5	20%	1	31.28	39.32	29.89
k_1_	51.65	54.71	54.04	49.30			
k_2_	52.17	47.58	45.12	47.17			
k_3_	39.15	40.68	43.81	46.51			
R	13.02	14.02	10.24	2.79			
k_1_	52.56	58.75	43.46	50.16			
k_2_	50.64	46.11	50.23	49.16			
k_3_	43.79	42.12	53.30	47.65			
R	8.77	16.63	9.84	2.51			
k_1_	40.26	45.29	33.03	39.08			
k_2_	39.43	35.36	37.54	37.37			
k_3_	33.92	32.97	43.04	37.17			
R	6.35	12.32	10.01	1.91			

**Table 4:** Extreme range analysis of the dynamic modulus.

No.	Orthogonal Factor				0 Cycles of Corrosion/GPa	60 Cycles of Corrosion/GPa	120 Cycles of Corrosion/GPa
	A	B	C	D (Empty)			
1	30%	0.3	0%	1	48.73	40.72	33.34
2	30%	0.4	20%	2	46.43	38.75	31.76
3	30%	0.5	40%	3	41.67	39.62	32.45
4	50%	0.3	20%	3	47.58	39.73	32.55
5	50%	0.4	40%	1	45.63	39.02	34.07
6	50%	0.5	0%	2	42.02	34.03	25.90
7	70%	0.3	40%	2	46.70	40.88	34.03
8	70%	0.4	0%	3	45.33	34.01	27.21
9	70%	0.5	20%	1	39.01	34.11	25.39
k_1_	45.61	47.67	45.36	44.46			
k_2_	45.08	45.80	44.34	45.05			
k_3_	43.68	40.90	44.67	44.86			
R	1.93	6.77	1.02	0.59			
k_1_	39.69	40.44	36.25	37.95			
k_2_	37.59	37.26	37.53	37.89			
k_3_	36.33	35.92	39.84	37.78			
R	3.36	4.52	3.58	0.16			
k_1_	32.52	33.30	28.81	30.93			
k_2_	30.84	31.01	29.90	30.56			
k_3_	28.88	27.91	33.52	30.74			
R	3.64	5.39	4.70	0.37			

**Table 5:** Analysis of variance of the compressive strength and dynamic elastic modulus.

Indicator	Corrosion Age	Source of Variance	Sum of Squared Deviations	Degrees of Freedom	Mean Square	F-Value	Critical Value Fa	Significance
Cubic compressive strength	0 cycle	Recycling aggregate replacement rate	325.50	2	162.75	25.82	F_0.1_(2.2) = 9	***
		Water–binder ratio	296.83	2	148.41	23.55	F_0.05_(2.2) = 19	***
		GGBS + fly ash content	186.99	2	93.49	14.83	F_0.01_(2.2) = 99	**
		Error	12.61	2	6.30			
		Total	21,213.76	9				
	60 cycle	Recycling aggregate replacement rate	127.46	2	63.73	13.32	F_0.1_(2.2) = 9	**
		Water–binder ratio	452.24	2	226.12	47.25	F_0.05_(2.2) = 19	***
		GGBS + fly ash content	152.18	2	76.00	15.90	F_0.01_(2.2) = 99	**
		Error	9.57	2	4.79			
		Total	22,345.27	9				
	120 cycle	Recycling aggregate replacement rate	71.42	2	35.71	10.81	F_0.1_(2.2) = 9	**
		Water–binder ratio	256.11	2	128.05	38.75	F_0.05_(2.2) = 19	***
		GGBS + fly ash content	150.88	2	75.44	22.83	F_0.01_(2.2) = 99	***
		Error	6.61	2	3.31			
		Total	13,392.36	9				
Dynamic modulus of elasticity	0 cycle	Recycling aggregate replacement rate	5.96	2	2.98	10.82	F_0.1_(2.2) = 9	**
		Water–binder ratio	73.32	2	36.66	133.11	F_0.05_(2.2) = 19	***
		GGBS + fly ash content	1.63	2	0.81	2.96	F_0.01_(2.2) = 99	*
		Error	0.55	2	0.28			
		Total	18,135.86	9				
	60 cycle	Recycling aggregate replacement rate	17.29	2	8.65	41.69	F_0.1_(2.2) = 9	***
		Water–binder ratio	32.37	2	16.19	782.01	F_0.05_(2.2) = 19	****
		GGBS + fly ash content	19.80	2	9.90	47.85	F_0.01_(2.2) = 99	***
		Error	0.04	2	0.02			
		Total	12,979.01	9				
	120 cycle	Recycling aggregate replacement rate	19.90	2	9.95	96.52	F_0.1_(2.2) = 9	***
		Water–binder ratio	43.90	2	21.95	212.93	F_0.05_(2.2) = 19	****
		GGBS + fly ash content	36.37	2	18.19	176.43	F_0.01_(2.2) = 99	****
		Error	0.21	2	0.10			
		Total	8606.59	9				

Note: “****” indicates highly significant. “***” indicates significant. “**” indicates a certain significance. “*” means insignificant.

**Table 6:** Regression equations for compressive strength and the dynamic elastic modulus.

Indicator	Corrosion Cycles/*n*	Regression Model	Correlation Coefficient (R^2^)
Cubic compressive strength	0 cycle	*y* = 96.49 − 31.25*x*_1_ − 70.33*x*_2_ − 25.67*x*_3_	0.84
	60 cycles	*y* = 88.29 − 21.92*x*_1_ − 83.14*x*_2_ + 24.61*x*_3_	0.91
	120 cycles	*y* = 65.44 − 15.87*x*_1_ − 61.61*x*_2_ + 25.03*x*_3_	0.9
Dynamic modulus of elasticity	0 cycle	*y* = 61.09 − 4.83*x*_1_ − 33.85*x*_2_ − 1.73*x*_3_	0.92
	60 cycles	*y* = 49.32 − 8.40*x*_1_ − 22.61*x*_2_ + 8.961*x*_3_	0.96
	120 cycles	*y* = 43.72 − 9.1*x*_1_ − 26.94*x*_2_ + 11.75*x*_3_	0.96

**Table 7:** Validation and error analysis of the compressive strength and dynamic elastic modulus regression models.

Corrosion Cycles	Replacement Ratio/%	Water-to-Binder Ratio	Fly Ash Content/%	Measured Compressive Strength/MPa	Measured Dynamic Elastic Modulus/GPa	Calculated Compressive Strength/MPa	Calculated Dynamic Elastic Modulus/GPa	Compressive Strength Error/%	Dynamic Elastic Modulus Error/%
0 cycle	30	0.3	0	66.73	48.73	66.02	49.49	−1.07	1.55
	30	0.4	20	48.55	46.43	53.85	45.76	10.91	−1.45
	30	0.5	40	39.68	41.67	48.72	42.02	22.77	0.85
	50	0.3	20	55.53	47.58	54.63	48.17	−1.62	1.25
	50	0.4	40	49.88	45.63	42.47	44.44	−14.87	−2.61
	50	0.5	0	51.09	42.02	45.7	41.75	−10.55	−0.64
	70	0.3	40	41.86	46.7	43.25	46.86	3.32	0.35
	70	0.4	0	44.31	45.33	46.48	44.17	4.90	−2.56
	70	0.5	2	31.28	39.01	34.32	40.44	9.71	3.66
60 cycles	30	0.3	0	57.95	40.72	56.77	40.02	−2.03	−1.72
	30	0.4	20	51.07	38.75	53.38	39.55	4.52	2.06
	30	0.5	40	48.65	39.62	49.99	39.08	2.74	−1.35
	50	0.3	20	60.29	39.73	57.31	40.13	−4.94	1.01
	50	0.4	40	53.22	39.02	53.92	39.66	1.31	1.65
	50	0.5	0	38.40	34.03	35.76	33.82	−6.88	−0.63
	70	0.3	40	58.02	40.88	57.85	40.24	−0.30	−1.56
	70	0.4	0	34.02	34.01	39.69	34.4	16.66	1.13
	70	0.5	20	39.32	34.11	36.3	33.92	−7.69	−0.57
120 cycles	30	0.3	0	44.04	33.34	42.2	32.91	−4.19	−1.28
	30	0.4	20	36.92	31.76	41.04	32.56	11.17	2.54
	30	0.5	40	39.82	32.45	39.89	32.22	0.16	−0.71
	50	0.3	20	45.82	32.55	44.03	33.44	−3.91	2.74
	50	0.4	40	43.30	34.07	42.87	33.09	−0.99	−2.86
	50	0.5	0	29.18	25.90	26.70	25.7	−8.51	−0.76
	70	0.3	40	46.00	34.03	45.86	33.97	−0.30	−0.18
	70	0.4	0	25.86	27.21	29.69	26.57	14.80	−2.33
	70	0.5	20	29.89	25.39	28.53	26.63	−4.53	4.87

## Data Availability

The original contributions presented in this study are included in the article. Further inquiries can be directed to the corresponding author.
